# Kinetics of Colour Development during Frying of Potato Pre-Treated with Pulsed Electric Fields and Blanching: Effect of Cultivar

**DOI:** 10.3390/foods10102307

**Published:** 2021-09-28

**Authors:** Setya Budi Muhammad Abduh, Sze Ying Leong, Chun Zhao, Samantha Baldwin, David J. Burritt, Dominic Agyei, Indrawati Oey

**Affiliations:** 1 Department of Food Science, University of Otago, Dunedin 9054, New Zealand; setya.abduh@live.undip.ac.id (S.B.M.A.); sze.leong@otago.ac.nz (S.Y.L.); p.zhao@otago.ac.nz (C.Z.); dominic.agyei@otago.ac.nz (D.A.); 2 Department of Food Technology, Universitas Diponegoro, Semarang 50275, Indonesia; 3 Riddet Institute, Palmerston North 4442, New Zealand; 4 The New Zealand Institute for Plant and Food Research Limited, Lincoln 7608, New Zealand; samantha.baldwin@plantandfood.co.nz; 5 Department of Botany, University of Otago, Dunedin 9054, New Zealand; david.burritt@otago.ac.nz

**Keywords:** kinetic, pulsed electric field, blanching, potato, cultivar, frying, colour, lightness, first order, activation energy, Arrhenius

## Abstract

The current research aimed to investigate the effect of pulsed electric fields (1 kV/cm; 50 and 150 kJ/kg) followed by blanching (3 min., 100 °C) on the colour development of potato slices during frying on a kinetic basis. Four potato cultivars ‘Crop77’, ‘Moonlight’, ‘Nadine’, and ‘Russet Burbank’ with different content of glucose and amino acids were used. Lightness (L* values from colorimeter measurement) was used as a parameter to assess the colour development during frying. The implementation of PEF and blanching as sequential pre-treatment prior to frying for all potato cultivars was found effective in improving their lightness in the fried products. PEF pre-treatment did not change the kinetics of L* reduction during frying (between 150 and 190 °C) which followed first-order reaction kinetics. The estimated reaction rate constant (*k*) and activation energy (*E_a_* based on Arrhenius equation) for non-PEF and PEF-treated samples were cultivar dependent. The estimated *E_a_* values during the frying of PEF-treated ‘Russet Burbank’ and ‘Crop77’ were significantly (*p* < 0.05) lower (up to 30%) than their non-PEF counterparts, indicating that the change in *k* value of L* became less temperature dependence during frying. This kinetic study is valuable to aid the optimisation of frying condition in deep-fried potato industries when PEF technology is implemented.

## 1. Introduction

Pulsed electric field (PEF) processing applies a high-voltage electric field in the form of short pulses (in the range of microseconds to milliseconds) across biological cells or foods to develop high transmembrane potential of the cells, leading to pore formation in the cell membrane and hence changes in the cell microstructure [1,2]. In the deep-fried potato industry, PEF processing is conducted as an intermediate step before cutting/slicing, blanching, pre-drying, and frying [3]. Implementation of PEF prior to cutting and blanching has been shown to be effective to soften potato tuber tissues and improve their processing textural quality leading to smoother cutting surface (i.e., reduced feathering) [4,5], reduction in starch loss [4], reduction in oil uptake [4,5] and a more uniform colour development during frying [5], as well as a reduction in acrylamide formation of the final deep-fried potato products [6]. Previous studies have reported the effectiveness of PEF in improving the quality of the final fried potato product. However, these studies conducted PEF treatments on a single potato cultivar available at the time of the experiment (e.g., ‘Agria’, ‘Rooster’, ‘Fontane’, and ‘Lady Claire’) [4,5,6]. This raises a question as to whether the impact of PEF treatment, when applied at the same process intensity, is consistent across potato cultivars with different physicochemical characteristics in terms of carbohydrate (such as sugar and starch), protein, dry matter, and water contents [7]. So far, no previous study has included the cultivar effect in PEF experimental designs conducted under similar process intensities to assess its impacts on colour development when PEF-treated potatoes are fried. Such study is indispensable for commercial deep-fried potato production since switching or blending potato cultivars with different physicochemical characteristics before entering the cutter/slicer is rather a common practice [8].

Blanching is one of the important unit operations that cannot be omitted during the production of deep-fried potato products because this process results in starch gelatinisation as well as microstructural changes in the potato tissues by weakening the binding between cells (i.e., middle lamella), leading to effective removal of glucose in the potato and hence minimising the development of undesirable dark brown colour in fried potato [9,10] that may lead to acrylamide formation [11]. This thermal process is also necessary to inactivate endogenous polyphenol oxidase enzyme responsible for the enzymatic browning in potatoes [12] and to precondition the tissue structure, enabling texture developments such as hardness and crispiness during frying [13]. Therefore, the adoption of PEF in a commercial potato processing line does not exclude blanching from the entire processing line, except for potato chips/crisps processing line that is using kettle or batch frying technique. Currently, kinetic information on colour development during frying is very limited especially for potatoes that have been subjected to PEF treatment followed by blanching.

The aim of this research was to study the kinetics of colour development during frying of potato slices from different potato cultivars after a sequential pre-treatment of PEF and blanching. In this study, four potato cultivars ‘Crop77’, ‘Moonlight’, ‘Nadine’, and ‘Russet Burbank’ were selected because they had considerable difference in inherent physicochemical properties and cooking quality or purpose (see Section 2.1). The kinetic rate constant (*k* value) and activation energy (*E_a_* value) of colour changes were predicted on the basis of first order reaction to assess the effect of cultivars and PEF pre-treatment applied to the potatoes prior to frying. This thorough kinetic study is a prerequisite to understanding the colour development of potato slices as a function of frying temperature and time. This approach is crucial to aid optimisation of frying conditions after conducting sequential PEF and blanching pre-treatment.

## 2. Materials and Methods

### 2.1. Raw Material and Characterisation of Their Chemical Composition

Four potato (*Solanum tuberosum* L.) cultivars, namely ‘Crop77’, ‘Moonlight’, ‘Nadine’, and ‘Russet Burbank’ (50 kg of tubers for each cultivar) were selected in this study. ‘Nadine’ is a common and readily available cultivar with waxy texture suitable for boiling, salads and braises, mainly sold as a fresh market washed potato in New Zealand [14]. ‘Moonlight’ is known as an all-rounder or general-purpose potato cultivar, in which the tubers are neither too floury not waxy, mainly grown for the local French fries industry as well as sold as fresh market potatoes [15]. ‘Russet Burbank’ is mainly used for the French fries industry [16]. ‘Crop77’ is a breeding line from the Plant and Food Research breeding programme specifically for potato crisping industry [17]. After harvest, the tubers from each cultivar were packed in 10 kg jute sacks and placed in a well-ventilated and temperature-controlled dark room at 8.8 ± 0.5 °C until use. Prior to processing, the content of total starch, amylose, glucose, total amino acids, and dry matter of the tubers was determined (see Supplementary Materials File S1).

### 2.2. Pulsed Electric Fields (PEF) Treatment

For each PEF experiment, potato tubers of similar size from the same cultivar were removed from the temperature-controlled room and acclimatised at room temperature (20 °C) overnight, before washing under running tap water to remove adhering soil. The skin of tubers was then peeled manually using a stainless-steel hand peeler. To reduce biological variation between and within tubers for experiment, the tubers were cut transversely into equal halves, separating the stem end from the basal end. Half of the amount of stem ends and basal ends were then allocated for control (later referred to as “Non-PEF”) and the remaining amount for PEF treatment (later referred to as “PEF-treated”).

The halved tuber was placed in a 400 mL PEF treatment batch chamber (100 mm length, 50 mm depth) consisting of two parallel stainless-steel electrodes (5 mm thickness, 80 mm electrode gap) with the cut surface facing the bottom of the PEF treatment chamber. Subsequently, the chamber was filled with sodium phosphate buffer (10 mM, pH 7.0, electrical conductivity of 1.40 ± 0.01 mS/cm at 13.77 ± 8.72 °C, prepared from appropriate mixture of monosodium dihydrogen orthophosphate and disodium hydrogen phosphate from Merck (Darmstadt, Germany)) as an electrical transferring medium, until the potato sample was fully immersed. The total weight of the potato sample and the medium was standardised for every PEF treatment (averaged at 316.97 ± 13.19 g).

Subsequently, potato samples were treated using an ELCRACK^®^ HVP 5 PEF system (German Institute of Food Technologies, Quakenbruck, Germany) at a fixed electric field strength of 1 kV/cm, 20 µs pulse width (square-wave bipolar pulses), 50 Hz pulse frequency at two different levels of specific energy inputs, i.e., approximately 50 kJ/kg (hereafter referred as “PEF Low”) and 150 kJ/kg (hereafter referred as “PEF High”). Equation (1) was used to calculate the specific energy input applied during PEF treatment. Considering the dimension of potato used in this study (i.e., halved tuber per PEF treatment to mimic industrial handling of potatoes), these two energy inputs were selected to achieve moderate to severe changes in textural properties based on our preliminary studies. This was consistent with other root vegetable studies on whole/halved potato [18], sweet potato [19] and carrot [20]. While previous studies have employed a much lower energy (<10 kJ/kg) on potato in the form of cubes (10–20 mm^3^) [5,21], cylinders (20–40 mm diameter, 4–10 mm height) [22,23,24], discs (9 mm thickness) [25] and slices (2.5 mm thickness) [26] to achieve effective cell disintegration, a higher energy input level is needed when handling whole or halved potato tubers.

(1) $$\mathrm{Specific}\;\mathrm{energy}\;\mathrm{input}\;(\mathrm{kJ}/\mathrm{kg})=\frac{V^{2}\;\cdot \;\left(\;n\;\cdot \;m\right)}{R\;\cdot \;W}$$

where *V* is pulse voltage (kV), *n* is pulse number (dimensionless), *m* is pulse width (µs), *R* is pulse resistance (ohm), *W* is the total weight of potato sample and electrical transferring medium in the PEF chamber.

The ELCRACK^®^ HVP 5 PEF system was equipped with built-in measurement sensors that allowed electric field strength (kV/cm), pulse voltage (kV), pulse current (A), pulse power (kW), pulse energy (J), total energy (kJ), pulse resistance (ohm) and pulse number to be monitored in real time during each PEF treatment. The pulse shape and voltage were monitored using a digital oscilloscope (UNI-Trend UTD2042C, Guangzhou, China). The temperature and electrical conductivity of the medium were measured before and after PEF treatment using a temperature/conductivity meter (CyberScan CON 11, Eutech Instruments, Singapore). The average temperature and conductivity increase after “PEF Low” were 6.07 ± 0.94 °C and 2%, respectively, while those after “PEF High” treatment were 15.09 ± 1.48 °C and 5%, respectively. For each potato cultivar, three independent PEF experiments were performed for “PEF Low” and “PEF High” treatments with each experiment using 8 to 10 potato tubers, where half the amount of stem and basal ends were used for PEF treatment and the remaining half as untreated samples. The total contact time between potato samples and sodium phosphate buffer during PEF treatment (starting from sample immersion in buffer solution in the PEF chamber to the completion of PEF treatment) was averaged at 2 min. For this reason, all the untreated potatoes (“Non-PEF”) were immersed in the sodium phosphate buffer for at least 2 min.

### 2.3. Kinetic Study on the Colour Changes of Non-PEF and PEF-Treated Potato Slices during Frying

#### 2.3.1. Kinetic Frying Experiment

The potato frying experiment was conducted using an electric fryer (Blue Seal E44E, Birmingham, UK). The fryer was filled with 15 L canola oil (BidFood Smart Choice 19540, Dunedin, New Zealand) and pre-heated for at least 1 h before frying. For each kinetic frying experiment, at least 8 to 10 potato tubers were prepared and then PEF-treated with “PEF Low” and “PEF High” treatments, as described earlier in Section 2.2. After each PEF treatment, all potato tubers were sliced to approximately 1.0 mm thickness using a mandolin (Benriner, Yamaguchi, Japan). Potato slices from different tubers within the same treatment group and cultivar were then pooled together and blanched in boiling distilled water for 3 min over an induction cooker (Micasa MA0239IC, Auckland, New Zealand). After blanching, the excess surface water of the potato slices was reduced using a food dehydrator (Sunbeam DT5600, Auckland, New Zealand) at 75 °C for 10 min. Then, the potato slices were immediately used for the kinetic frying experiment.

At each frying temperature, approximately 50 slices of potato (±150 g) were randomly selected and transferred into a stainless-steel frying basket. When the oil reached the targeted temperature as monitored using a digital thermometer (Breville BMP100, Sydney, Australia), the frying basket was immersed in the hot oil. Up to 4 potato slices from ‘Russet Burbank’, ‘Nadine’ and ‘Moonlight’ were removed from the fryer at every time intervals of 60 s, 40 s, and 30 s for frying temperatures of 150, 170, and 190 °C, respectively. Our preliminary study showed that the colour of ‘Crop77’ potatoes changed very minimal when being fried for up to 15 min due to the exceptional low amount of glucose for this cultivar (Table 1). Therefore, the standard protocol for Nadine, Moonlight and Russet Burbank was adapted for ‘Crop77’ by frying at 170, 180 and 190 °C to allow accurate estimation of *k* and *E_a_* values. For these reasons, potato slices from ‘Crop77’ were deep-fried at higher temperatures of 170, 180 and 190 °C to better study the changes in their colour as a function of frying time. The temperature-time profile of potato slices during frying was monitored using a K type thermocouple (0.2 mm diameter; Labfacility, South Yorkshire, UK) and recorded using a data logger (Picotech TC-08, Cambridgeshire, UK). As soon as the fried potatoes were removed from the fryer, they were placed on a paper towel to absorb excess surface oil and cooled. The kinetic frying experiment was repeated 3 times for each cultivar, where at least 8 to 10 potato tubers were used for each independent replication.

The CIE L*, a* and b* colour of the fried samples was measured using a pre-calibrated colourimeter (MiniScanEZ-4500L, Hunterlab, Reston, VA, USA) based upon tristimulus CIE colour combination values, i.e., L* (lightness (100) to darkness (0)), a* (red (+) to green (−)), and b* (blue (+) to yellow (−)) under D65-artificial daylight at 10° standard angle. Due to the uneven surface of the fried potato slices, each sample was crushed finely using a bread roller and transferred into a white porcelain cup for colour measurement. A total of three colour measurements were taken for each fried sample.

#### 2.3.2. Estimation of the Time Dependence of the Colour Change of Potato Slices during Frying

The colour development of potato slices during frying typically followed first-order reaction kinetics (Equation (2)), as reported in other potato cultivars namely ‘Panda’ based on b* value [27], ‘Desiree’ based on total colour difference (Δ*E*) [28,29], ‘Rosetta’ based on L*, a*, and b* values [30] and ‘Russet Burbank’ based on L*, a*, b*, and Δ*E* values [31].

In this study, L* (lightness) value was used to describe the colour change of potato slices from all four cultivars, with and without PEF and blanching pre-treatments, during frying as L* was the only parameter that best fitted to the linearised form of the first-order model (Equation (3)) with R^2^ close to 0.9. The rate constant to describe changes in the L* value (*k*, in s^−1^) at each frying temperature was then estimated on the basis of Equation (3) [32].

(2) $$L^{*}{=L}_{0}^{*}\;\cdot {\;e}^{-k\;\cdot \;t}$$

(3) $${\ln L}^{*}{=\ln L}_{0}^{*}-\;k\;\cdot \;t$$

where L* is the lightness of potato slices at frying time of *t* (dimensionless), L*_0_ is the lightness of potato slices at time *t* = 0 s, *k* is the rate constant (s^−1^) for changes in the L* value during frying, and *t* is frying time (s). The rate constant of lightness change (*k*) at each frying temperature was estimated as the slope from the plot of the natural logarithm of lightness (L*) against frying time (*t*). Microsoft Excel (Microsoft Corporation, Redmond, WA, USA) was used to estimate *k* values (based on Equation (3)) for non-PEF and PEF-treated samples fried at each frying temperature for three independent kinetic frying experiments.

#### 2.3.3. Estimation of the Temperature Dependence of Rate Constant for Changes in L* Value during Frying

The temperature dependence of the rate constant *k* for changes in L* value during frying was estimated using Arrhenius equation (Equation (4)), which can be linearised using a logarithmic transformation, leading to Equation (5) [33].

(4) $$\;k=A\cdot \exp\left(\frac{-E_{a}}{R\cdot \;T}\right)$$

(5) $$\ln k=\ln A-\frac{E_{a}}{R\cdot \;T}$$

where *k* is the rate constant (s^−1^) for changes in L* value at a specific frying temperature as estimated from Equation (3), *A* is a pre-exponential factor with the same dimension as that of *k*, *E_a_* is the activation energy (kJ/mol^−1^), *R* is the universal gas constant (8.314 J∙mol^−1^∙K^−1^), and *T* is the actual frying temperature (K). The kinetic parameter *E_a_* was estimated using a linear regression analysis by plotting the natural logarithm of the rate constant (*k*) of lightness (L*) change versus the reciprocal of the absolute temperature (1/*T*) using Microsoft Excel (Microsoft Corporation, Redmond, WA, USA).

### 2.4. Statistical Analysis

The statistical significance of difference in the chemical content and colour parameters of potato samples between cultivars or between treatments was calculated using Student’s *t*-test for single comparison or using an analysis of variance (ANOVA) for multiple comparisons, followed by post hoc Tukey’s Honestly Significant Difference (HSD) test. Pearson’s correlation coefficient (*r*) was used to determine the linear correlation between chemical content in potato tuber and the colour parameters of fried samples, followed by evaluation of statistical significance of the linear correlation with two-tailed probability values. The criterion employed for a statistical significance of the difference was *p* < 0.05. All statistical analysis was performed using IBM SPSS Statistics version 25 (IBM Corporation, New York, NY, USA).

## 3. Results and Discussion

### 3.1. Comparison on the Chemical Composition of Four Different Potato Cultivars

Table 1 summarises the dry matter, total starch, amylose, glucose and total amino acid contents of the four potato cultivars used in this study. Dry matter of potato tubers varied significantly (*p* < 0.05) between the four cultivars, with both ‘Crop77’ and ‘Russet Burbank’ had the highest dry matter (>24%) and the lowest in ‘Nadine’ (<15%). Dry matter is known to be associated with the texture of potato when fried, where potatoes rich in dry matter usually resulted in a crispier texture after frying compared to those produced using potato cultivars with a lower dry matter [34]. Starch is a major component in potato, and it was found that most potato cultivars used in this study had similar total starch content (>708 mg/g DW), except for ‘Moonlight’ with the lowest total starch content, averaged at 587.34 mg/g DW. Moreover, it was found that amylose made up at least 24% of the total starch content in ‘Moonlight’ and ‘Nadine’, while amylose only constituted at least 10% of the total starch content for ‘Crop77’ and ‘Russet Burbank’. Previous studies have shown that differences in the amylose content between potato cultivars is not uncommon and can influence the functional properties of starch, cooking quality and its end use for food application [35,36].

Glucose is an important precursor involved in the colour development of potato during frying, i.e., through caramelisation and the Maillard reaction [30]. When comparing the glucose content between the potato cultivars, ‘Nadine’ displayed the highest levels of glucose (104.64 mg/g DW), followed by ‘Moonlight’ and ‘Russet Burbank’ sharing similar range between 21 and 22 mg/g DW, and it was interesting to detect a very low amount of glucose in ‘Crop77’ (0.75 mg/g DW) (Table 1). Therefore, it is expected that the high glucose content in ‘Nadine’ will lead to excessive colour browning in the potato slices when fried. According to Bartlett, et al. [37], potato cultivar with total reducing sugars of greater than 50 mg/g is generally considered as tubers with a high acrylamide risk in any deep-fried potato products manufacturing line (e.g., French fries and potato crisp). Another key precursor involved in Maillard reaction to allow reaction with the carbonyl group of glucose is amino acid [30]. In this study, it was found that the total amino acid contents of ‘Nadine’, ‘Russet Burbank’ and ‘Moonlight’ were similar and were significantly (*p* < 0.05) higher than that of ‘Crop77’.

Clearly, ‘Crop77’ potatoes were characterised by low glucose content, low in amino acids but high in dry matter and total starch. ‘Russet Burbank’ and ‘Moonlight’ potatoes shared a few similarities in the chemical contents, where both cultivars had moderate levels of glucose and amino acids. However, the starch content in ‘Russet Burbank’ was higher than in ‘Moonlight’. High glucose and amino acids contents, in conjunction with low dry matter are the chemical features for ‘Nadine’ cultivar. With this in mind, ‘Crop77’ and ‘Nadine’ represent the low- and high-glucose control potatoes in this study, respectively. It is, however, important to note that the use of ‘Nadine’ tubers for deep-fried potato processing line is typically unfavourable due to its exceptionally high glucose content and low dry matter.

### 3.2. Colour Evaluation of Fried Potato Slices Produced from Non-PEF and PEF-Treated Potatoes

To justify the effectiveness of PEF in controlling the colour development of potato slices during frying, the inclusion of low- and high-glucose control potatoes (i.e., using ‘Crop77’ and ‘Nadine’ respectively in this study) is considered for the first time in the literature. The colour characteristics of potato slices fried under the same frying condition i.e., at 180 °C for 3 min, without PEF nor blanching pre-treatments were initially assessed. In agreement with the high glucose content found in ‘Nadine’, it is not unexpected that the resulting fried samples from this cultivar displayed the lowest L* (reduced lightness) and b* (reduced yellowness) (Table 2) compared to other potato cultivars, when all fried at 180 °C for 3 min. On the contrary, potato slices from ‘Crop77’, with the glucose content averaged at least 140-fold lower than ‘Nadine’, exhibited the highest L* (lightness was at least 2-fold higher than fried ‘Nadine’), lowest a* (redness was at least 1.3-fold lower than fried ‘Nadine’), and the highest b* (yellowness was at least 2-fold higher than fried ‘Nadine’) when fried (Table 2). The average a* values for fried potato slices from ‘Moonlight’ and ‘Russet Burbank’ were rather similar (Table 2) and significantly redder compared to fried ‘Nadine’ and ‘Crop77’. While both ‘Russet Burbank’ and ‘Moonlight’ shared similar glucose content (Table 1), ‘Russet Burbank’ fried samples were found to demonstrate a higher average L* and lower b* value compared to fried samples from ‘Moonlight’. Based on the Pearson’s correlation analysis, the difference in the colour characteristics of fried potato slices (Table 2) from four cultivars, especially L* (*r* = −0.9893) and b* (*r* = −0.9810), appeared to correlate significantly (*p* < 0.05) with the level of glucose (i.e., one of the important precursors involved in the Maillard reaction browning during frying) found in the tubers (Table 1). The a* value of fried potato slices was found to be positively correlated with the total amino acids content in the tuber instead (*r* = 0.8769, *p* < 0.05). Coincidentally, previous studies have consistently evidenced that both L* and b* values of fried potatoes are highly associated with the glucose content of potatoes [7], while the colour parameter a* correlates better with amino acids and acrylamide concentration [38].

#### 3.2.1. The Effect of PEF Pre-Treatment Alone (without Blanching) on the Colour Characteristics of Fried Potato Slices

Based on Table 2, the colour characteristics of fried potatoes was found to be highly dependent on the initial glucose content of the tubers and the intensity of PEF treatment. Fried ‘Nadine’ slices produced from PEF Low treatment exhibited a higher lightness (L*) and reduced redness (a*) compared to fried samples from untreated ‘Nadine’. Moreover, a* was the only colour parameter for fried ‘Russet Burbank’ slices, produced from PEF Low treatment, found to be reduced significantly (*p* < 0.05) when compared to untreated counterpart. Therefore, the results indicate that application of PEF Low pre-treatment on the tubers prior to frying is particularly effective for high glucose-containing ‘Nadine’ to limit the degree of Maillard browning upon frying. The cell electroporation effect of PEF on promoting the leakage of chemical constituents [39] from potato tuber is likely the reason to minimise the level of precursors (e.g., sugars) involved in Maillard reaction. With respect to the ‘Crop77’ and ‘Moonlight’, the application of PEF Low treatment on the tubers did not influence both the lightness and redness of fried potato slices. Therefore, it can be concluded that an application of PEF Low pre-treatment on potatoes was promising in controlling the colour development during frying when applied on a high glucose-containing potato cultivar such as ‘Nadine’ and posed almost negligible effect when applied on a low glucose-containing potato cultivar such as ‘Crop77’. This is because the glucose content in ‘Nadine’ potatoes was reduced by 74% with PEF Low pre-treatment while no apparent changes in the glucose content for ‘Crop77’ potatoes before and after PEF treatment. PEF treatment tends to cause changes to the microstructure of plant material and a recent study by Zhang et al. [40] showed that potato cells can be severely disrupted by PEF in which larger pores can then be formed, accelerating the mass transfer process such as leakage of cell constituents after PEF.

Similar to PEF Low treatment, the application of PEF at higher energy (i.e., increased from 50 to 150 kJ/kg at a fixed electric field strength of 1 kV/cm) did not cause significant changes in the colour parameters of the fried ‘Crop77’ slices. The reason is due to the exceptional low glucose content for this potato cultivar as no considerable in glucose reduction was observed after PEF. If leaching of glucose from the ‘Crop77’ potato slices did occur due to PEF, the amount of glucose remained in the tissue could be too low to influence the colour characteristics of fried potato slices. On the contrary, it was found that PEF High treatment significantly (*p* < 0.05) improved the colour parameters for both ‘Russet Burbank’ (a* reduced from 5.6 to 4.2) and ‘Nadine’ (L* increased from 17 to 20 and a* reduced from 4.5 to 3.5) fried samples to that of their untreated counterparts. However, it is important to note the extent of improvement in lightness and reduction of redness of fried samples for these two cultivars by PEF High pre-treatment were similar as when PEF Low treatment was applied to these tubers prior to frying. This result strongly suggested that application of PEF at an electric field strength of 1 kV/cm and energy ~50 kJ/kg on potatoes was adequate in controlling the degree of colour changes upon frying.

#### 3.2.2. The Effect of Sequential PEF and Blanching Pre-Treatment on the Colour Characteristics of Fried Potato Slices

When the potato slices were blanched and then fried immediately at 180 °C for 3 min, fried samples from all non-PEF potatoes, except for ‘Crop77’, exhibited a significant (*p* < 0.05) improvement in lightness (L*) compared to their unblanched counterparts (Table 3). The extent of L* increase was the greatest for fried ‘Nadine’, followed by ‘Moonlight’ and ‘Russet Burbank’. The fact that fried ‘Nadine’ exhibited the greatest increase in L* indicates that blanching is a vital unit operation to effectively remove sugars from the potato of inherently high sugar load to reduce the excessive colour development of fried potato slices associated with Maillard reaction. In this study, blanching was able to cause up to 66% reduction in glucose in ‘Nadine’. Blanching pre-treatment also led to a significant (*p* < 0.05) reduction in redness (a*) of fried samples for all potato cultivars, except ‘Nadine’. With respect to the yellowness (b*) of the fried potato slices, the impact of blanching treatment was not consistent between cultivars. Compared to their unblanched counterparts, b* reduced significantly for blanched ‘Crop77’, increased significantly for both ‘Russet Burbank’ and ‘Nadine’, and no changes in b* was observed for fried samples between unblanched and blanched ‘Moonlight’. Despite this, the b* value of fried potato slices from all 4 cultivars appeared to fall within the same range (between 12.88 and 18.03), indicating that the variation in b* of fried samples between cultivars was minimised with blanching pre-treatment and such observation was not found in other colour parameters of L* and a* of fried potato samples.

Moreover, the results from this study showed that the lightness of fried ‘Russet Burbank’ slices with blanching pre-treatment overlapped with ‘Crop77’ and ‘Moonlight’, which was not exhibited in their unblanched counterparts. With respect to the redness of fried potato slices, unblanched ‘Moonlight’ and ‘Russet Burbank’ upon frying exhibited the highest a* among all four cultivars (Table 2) and the subsequent application of blanching step reduced their redness significantly (Table 3). Clearly, blanching can reduce the colour development of fried potato slices and possibly minimise variation in colour across different potato cultivars of varying levels of glucose due to the effective removal of inherent sugars prior to frying. In fact, recent studies by Bartlett, et al. [37] and Zhang, et al. [41] consistently reported that extending the blanching duration further provoked changes in the microstructure of potato tissues, consequently facilitating the release of sugars and amino acids involved in Maillard reaction, and thus maximising the reduction of acrylamide of potato slices after frying.

The sequential PEF and blanching treatment on the colour characteristics of fried potato slices is presented in Table 3. When PEF Low was applied to the potatoes, followed by blanching, the L* of resulting fried samples improved significantly (*p* < 0.05) for those produced from ‘Crop 77′ and ‘Russet Burbank’. Such observation was not found in those fried samples when the potatoes were treated with PEF Low alone without subjected to the subsequent blanching step (Table 2). In particular, the subsequent blanching step after PEF Low treatment further increased the lightness of fried potato slices of low glucose-containing ‘Crop 77′. Furthermore, the redness of fried samples reduced significantly (*p* < 0.05) for all cultivars after the sequential PEF Low and blanching treatment compared to their non-PEF counterparts, while the yellowness of the resulting fried samples for all potatoes were similar to their non-PEF counterpart. Among all the cultivars, the sequential PEF Low and blanching treatment was found effective in causing between 22 (least in ‘Crop77’) and 74% (the greatest in ‘Nadine’) reduction in glucose. In this context, the effective removal of glucose from potato tissues could be attributed by the cell electroporation effect after PEF combined with the depolymerisation and solubilisation of pectin during blanching step [42].

When the sequential PEF High and blanching treatment applied to the potatoes, only fried ‘Russet Burbank’ samples experienced a significant (*p* < 0.05) increase in L* compared to non-PEF counterparts. However, the lightness of fried ‘Russet Burbank’ was similar when the tubers were treated with PEF Low and PEF High, suggesting that the application of sequential PEF Low and blanching treatment was adequate to improve the lightness of potato slices upon frying. A similar result was observed with respect to the redness and yellowness of fried samples. The glucose result shows that the glucose amount remaining in ‘Russet Burbank’ was similar when pre-treated with either PEF Low or PEF High treatment. However, the sequential PEF High and blanching treatment on ‘Moonlight’ appeared effective in reducing the yellowness of the resulting fried samples (Table 3). The reason behind could be due to the ability of sequential PEF High and blanching treatment in causing up to 80% reduction in glucose, which has not been observed in any other cultivars.

Overall, it is interesting to observe that the colour characteristics (especially L* and a*) of fried potato slices for all cultivars were impacted more significantly due to sequential PEF and blanching treatments compared to when PEF treatment alone (without blanching step) was applied. Therefore, findings from this study revealed a positive synergistic effect of the sequential PEF and blanching treatment on potato, when tested across four potato cultivars with varying content of inherent glucose and amino acids, in improving the colour characteristics of fried potato slices. The result also showed that PEF treatment could be possibly applied at a lower intensity by combining with blanching step during the production of fried potato slices to achieve improved colour characteristics. For example, fried ‘Russet Burbank’ and low glucose-containing ‘Crop77’ from any PEF-treated tubers shared similar lightness. Due to consistent and significant improvements in the colour characteristics of fried potato slices observed across four cultivars, the effect of PEF followed by blanching treatments on the kinetics of their colour changes during frying was modelled and is further discussed in the next section (Section 3.3). The inclusion of combined PEF as a pre-treatment with a subsequent blanching step is considered to be a more effective and strategic acrylamide mitigation measure when producing deep-fried potato products [43].

### 3.3. Kinetic Study on the Colour Changes of Sequential PEF and Blanching Pre-Treated Potatoes during Frying

The Maillard browning reaction is a complex reaction, and colour development is considered the final phase in this reaction [32]. The first-order kinetic model has been applied on the initial, intermediate, and final phases of the Maillard reaction [33]. Therefore, in this study the change in colour parameters L*, a* and b* of pre-blanched potato slices for all four cultivars were modelled as a function of frying time and temperature. Compared to other colour parameters, the changes in L* value followed a first-order reaction, and the measured data fitted well to the first order reaction equation (Equation (3)) with *R*^2^ close to 0.9. Therefore, the L* parameter was used in this study to describe the colour development of potato slices during frying.

#### 3.3.1. Time and Temperature Dependences of L* Value Change for Non-PEF (Blanching Only) Pre-Treated Potatoes during Frying

**Figure 1:**
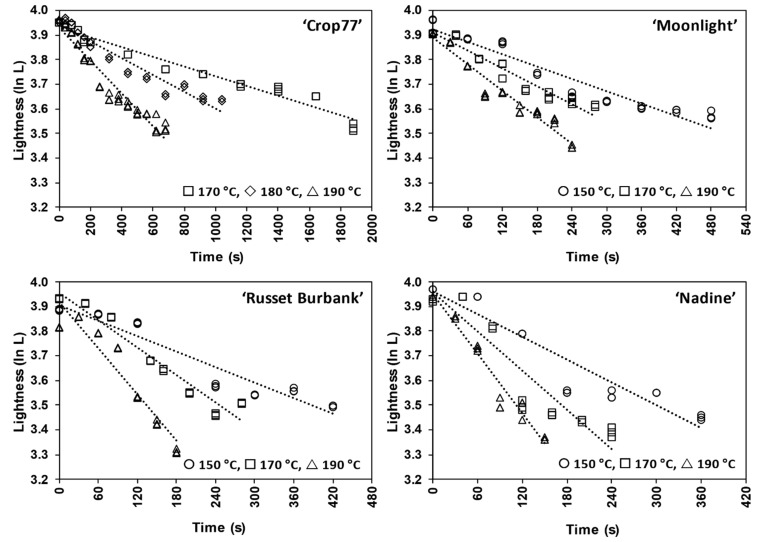
An example of fitting the first-order kinetic model to the experimental lightness data (natural logarithm of L* values against frying time) at different frying temperatures for ‘Crop77’, ‘Moonlight’, ‘Nadine’ and ‘Russet Burbank’ subjected to blanching treatment only. Symbols represent the experimental lightness data (average measurements from three independent frying experiments per frying time point where each experiment involved a batch of 8–10 potatoes) and the predicted lightness values from the first-order reaction (Equation (3)) are represented by the continuous dots. The L* of potato slices prior to frying was averaged at 57.15 ± 1.33, 57.09 ± 1.48, 54.60 ± 2.17 and 52.76 ± 1.49 respectively for ‘Crop77’, ‘Moonlight’, ‘Nadine’ and ‘Russet Burbank’.

Representative plots of the natural logarithm of L* (ln L*) against frying time, for fried potato slices from the four cultivars and the fitting of the first-order kinetic model to the experimental lightness data, are illustrated in Figure 1. Clearly, changes in the lightness for all investigated cultivars during frying obeyed first order reaction kinetics (Equation (3)). The rate constant (*k*) describing the changes of L* at each frying temperature was estimated using linear regression analysis (Table 4). The estimated rate constant *k* increased with increasing frying temperature, indicating the rate of changes in lightness of potato slices typically accelerated when they are fried at higher temperatures. At the same frying temperature (i.e., 170 and 190 °C), there was an apparent cultivar effect on the estimated *k* values in L*, where the fried potato slices from ‘Nadine’ were found to darken (reduction in L*) the fastest (1.93–2.96 × 10^−3^ s^−1^), followed by ‘Russet Burbank’ (1.63–2.87 × 10^−3^ s^−1^) and ‘Moonlight’ (1.27–1.87 × 10^−3^ s^−1^), and the slowest in ‘Crop77’ (0.24–0.83 × 10^−3^ s^−1^). Moreover, findings from this kinetic study revealed that the estimated *k* value in L* for high glucose-containing ‘Nadine’, together with ‘Russet Burbank’ and ‘Moonlight’, fried at 150 °C are likely to fall into a similar rate as low glucose-containing ‘Crop77’, when fried at 190 °C (Table 4).

**Figure 2:**
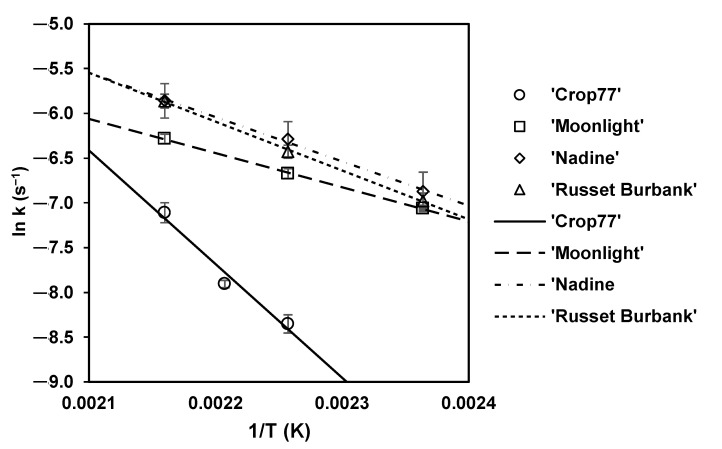
Arrhenius plot of the natural logarithm of *k* values for changes in L* during frying against the reciprocal of absolute frying temperature for ‘Crop77’, ‘Moonlight’, ‘Nadine’ and ‘Russet Burbank’ subjected to blanching pre-treatment only. Symbols represent the estimated *k* values for L* from first-order reaction (Equation (3)) and the standard error bars represent the standard deviation (average from three independent frying experiments where each experiment involved a batch of 8–10 potatoes).

To describe the temperature dependence of *k* values for the four potato cultivars, the Arrhenius equation (Equation (5)) was used. The result of fitting the Arrhenius equation to the natural logarithm of *k* (ln *k*) describing the change in L* for fried potatoes and the reciprocal of absolute temperature (1/T) is depicted (Figure 2). It was found that the estimated *E_a_* values varied significantly (*p* < 0.05) according to the potato cultivar (Table 4), ranging from 31.73 kJ/mol (lowest in ‘Moonlight’) to 105.53 kJ/mol (highest in ‘Crop77’). Moreover, the estimated *E_a_* values for the changes in lightness during frying of ‘Nadine’ potato slices was significantly (*p* < 0.05) lower than ‘Russet Burbank’. The estimated *E_a_* value obtained in this study for fried ‘Russet Burbank’ (45.37 kJ/mol) was slightly higher than previous study reported at 43.2 kJ/mol [31]. Potato cultivar displaying a lower estimated *E*_a_ values typically indicate a lower temperature dependency of the *k* values with respect to the lightness of fried samples, and vice versa for those potato cultivars displaying a higher estimated *E*_a_ values. Overall, the *k* values of colour parameter L* during the frying of ‘Moonlight’, ‘Nadine’ and ‘Russet Burbank’ potato slices appeared to be less temperature sensitive (or more temperature stable) than that of low glucose-containing ‘Crop77’ potatoes.

#### 3.3.2. Time Dependency of L* Value for PEF-Treated Potatoes during Frying

Table 4 summarises the rate constant (*k*) describing the reduction of L* increased with frying temperature, estimated for PEF-treated potatoes of different cultivars. Interestingly, when either PEF Low or PEF High were applied to the ‘Russet Burbank’ potatoes, a significantly (*p* < 0.05) lower *k* value for lightness in the fried potato slices at 190 °C was observed in comparison to their non-PEF (only subjected to blanching) counterparts. This observation strongly suggests that PEF-treated ‘Russet Burbank’ experienced a slower reduction in the L* colour parameter during frying at high temperature of 190 °C. It could be that the cell electroporation effect of PEF treatment, followed by the subsequent blanching step, modified the structural integrity of potato cells [42] and possibly altered the levels of precursors (e.g., reducing sugars and amino acids) responsible for Maillard browning reactions in the potato slices prior to frying, thus delaying the colour darkening (i.e., L* reduction) in the fried potato slices. Nevertheless, fried potato slices produced from the other three potato cultivars after sequential PEF and blanching treatments did not exhibit a similar trend in rate of colour change in L* to that of ‘Russet Burbank’ at any frying temperature. In other words, the estimated *k* values for changes in L* at each frying temperature for potato slices from low glucose-containing ‘Crop77’, ‘Moonlight’ and high glucose-containing ‘Nadine’ were not affected at a statistical significant level (*p* > 0.05) by any of the PEF pre-treatments applied (Table 4). This result implies that the effectiveness of sequential PEF and blanching treatments in reducing the rate of lightness during potatoes frying could be highly dependent on the cultivar. Apart from the differences in the availability of precursors for the colour development in Maillard reaction, variation in the solid content of potato has been reported to influence their tissue conductivity, thus impacting the efficacy of PEF treatment [22].

#### 3.3.3. Temperature Dependency of *k* for L* Value for PEF-Treated Potatoes during Frying

Table 4 shows the estimated activation energy (*E_a_*) describing the changes of L* for each potato cultivar during frying due to sequential PEF and blanching treatments. Regardless of the intensity of PEF pre-treatment applied, the fried samples from ‘Crop77’ consistently demonstrated the highest estimated *E_a_* value with respect to the changes in L* during frying. On the contrary, fried samples from any pre-treated ‘Moonlight’ consistently demonstrated the lowest estimated *E_a_* value.

The estimated *E_a_* values for the rate of L* changes of fried potato slices were significantly (*p* < 0.05) lower in all PEF-treated ‘Russet Burbank’ compared with the non-PEF samples (Table 4). A similar finding was found for fried samples from PEF-treated low glucose-containing ‘Crop77’ but not for ‘Moonlight’ and high glucose-containing ‘Nadine’. The *k* values describing the changes of colour parameter L* during the frying of ‘Russet Burbank’ and ‘Crop77’ potato slices appeared to be significantly (*p* < 0.05) more temperature stable (i.e., reduction of estimated *E_a_*) because of the application of sequential PEF and blanching treatment. This is contrary to the opposite behaviour exhibited by PEF-treated sweet potato during frying [19], where the estimated *E_a_* value describing the temperature dependency of the rate of changes of L* increased for PEF-treated sweet potato, suggesting the resulting slices become more temperature sensitive towards browning/darkening when fried. The discrepancy between the sweet potato result and the findings from the present study on potato tubers could be due to the tissue type difference between the two vegetable matrices [44], and also attributed to the fact that a blanching step was applied to all potatoes in the present study prior to frying.

Another interesting finding from this study is that the estimated *E_a_* values for ‘Crop77’ seem to demonstrate a PEF processing intensity specific effect, where a lower *E_a_* value was estimated for fried samples from ‘Crop77’ pre-treated at PEF Low compared to those treated at PEF High. This finding was not observed for ‘Russet Burbank’ as the *E_a_* values for fried potato slices from PEF Low-treated were significantly (*p* < 0.05) higher than PEF High-treated ‘Russet Burbank’ potato slices. Overall, potato slices from PEF-treated ‘Russet Burbank’ (especially at PEF High) and low glucose-containing ‘Crop77’ (especially at PEF Low) are expected to experience a small change in L* parameter (i.e., slower browning) during frying over a wider temperature range (i.e., more temperature stable).

The availability of glucose and amino acids on the surface of potato slice, as a reactant for the formation of melanoidin, as part of the Maillard reaction that led to browning of potato slices during frying, is highly associated with the diffusion characteristic of potato slices, the mass transfer of frying oil penetrating the potato slice, and moisture leaving the potato tissue during frying [45,46]. Pore formation at the cell membrane of potato tissue due to PEF treatment has been reported to cause changes in the diffusion characteristics of potatoes [22,23,39,47], thus affecting the removal of sugar and the subsequent heat and mass transfer processes during frying [48,49]. Moreover, the inclusion of a blanching step after PEF treatment, prior to frying, is another effective way of removing excess sugar from the potatoes by diffusion process due to severe disruption of potato tissues of the thermal effect of blanching in weakening the binding between cells (i.e., middle lamella) [10,42]. The aforementioned are likely the reasons that the estimated kinetic parameters underpinning colour development during potato slices frying was significantly altered for ‘Crop77’ and ‘Russet Burbank’ when both were subjected to sequential PEF and blanching treatments (Table 4). However, it is not possible to explain why similar results were not observed for ‘Moonlight’ and high glucose-containing ‘Nadine’.

## 4. Conclusions

This study provides an important insight with respect to the colour development of fried potato slices when potato tubers with significant inherent differences in the content of precursors involved in Maillard reaction browning, namely glucose and amino acids, were subjected to sequential PEF and blanching treatments prior to frying. PEF pre-treatment did not change the kinetics of changes in L* values during frying for any of the four cultivars, namely ‘Crop77’, ‘Moonlight’, ‘Nadine’ and ‘Russet Burbank’, which followed first-order reaction kinetics. While frying of potato slices of low glucose-containing ‘Crop77’ exhibited the highest *E_a_* value for changes in L* among all cultivars, it was found that the estimated *E_a_* value decreased significantly (i.e., more temperature stable), by at least 18% when the potatoes were pre-treated with PEF Low prior to frying. With respect to high glucose-containing ‘Nadine’, this cultivar is generally not suitable for commercial deep-fried potato process lines, but the findings from this study reveal that sequential PEF and blanching treatment is very effective at reducing the L* of ‘Nadine’ slices, with reductions of up to 38%. In addition, the frying kinetic result of non-PEF and PEF-treated ‘Nadine’ showed that the rate of changes in L* was less temperature sensitive than equivalently pre-treated low glucose-containing ‘Crop77’. In this study, it was rather surprising to find that potato slices from moderate glucose-containing ‘Moonlight’ exhibited the lowest estimated value of *E_a_* among all cultivars for any PEF pre-treatment applied to the tubers prior to frying, indicating the *k* value for changes in L* of the potato slices for this cultivar is less temperature sensitive with increasing frying temperature. The sequential PEF and blanching treatments appeared to benefit ‘Russet Burbank’ the most in which changes in L* of the potato slices became more temperature stable (i.e., up to 30% reduction in *E_a_* value with PEF High treatment) over a wide range of frying temperature when higher intensity of PEF was applied to the tuber prior to frying. Clearly, this research provides new evidence that colour development of potato slices during frying can be modulated with PEF pre-treatment on tubers in conjunction with smart selection of potato cultivar. The reduction of estimated *E_a_* value due to sequential PEF and blanching treatments was prominent, particularly when frying potato slices from ‘Crop77’ and ‘Russet Burbank’, which may bring advantages to the process control of deep-fried potato industries, especially when the temperature distribution inside the fryer is not uniform during processing. It is expected that the results of the present study would be helpful in predicting the impact of sequential PEF and blanching treatments on the colour development of potato tubers from a wide range of physicochemical properties.

## Figures and Tables

**Table 1:** Tuber characteristics, dry matter, total starch, amylose, glucose, and amino acid contents of ‘Crop77’, ‘Moonlight’, ‘Nadine’, and ‘Russet Burbank’ used in this study.

Tuber Characteristics and Chemical Contents	Potato Cultivar			
	‘Crop77’ ^†#^	‘Moonlight’ ^†^	‘Russet Burbank’	‘Nadine’ ^##^
Shape of tuber	Short-oval	Oval	Long	Oval
Colour of skin	Cream	Cream	Cream	Cream
Colour of flesh	White	White	White	Cream
Dry matter (%)	24.61 ± 1.30 ^a^	20.57 ± 0.60 ^b^	24.09 ± 0.51 ^a^	14.92 ± 0.38 ^c^
Total starch (mg/g DW)	731.04 ± 69.96 ^a^	587.34 ± 88.72 ^a^	708.95 ± 27.21 ^a^	722.87 ± 46.60 ^a^
Amylose (mg/g DW)	75.53 ± 7.84 ^b^	147.23 ± 5.38 ^a^	81.73 ± 3.61 ^b^	172.68 ± 10.17 ^a^
Glucose (mg/g DW)	0.75 ± 0.09 ^c^	20.68 ± 1.46 ^b^	22.35 ± 1.60 ^b^	104.64 ± 2.77 ^a^
Total amino acids (mmol/g DW)	0.99 ± 0.07 ^b^	1.78 ± 0.20 ^a^	1.64 ± 0.19 ^a^	1.64 ± 0.04 ^a^

The data are presented as mean ± standard error (*n* = 3–6). Means in the same row not sharing the same letter are significantly different at *p* < 0.05 between cultivars based on one-way ANOVA and Tukey’s post hoc test. DW = dry weight. ^†^ Originated from the breeding programme of the New Zealand Institute for Plant and Food Research Limited. ^#^ ‘Crop77’ represents the low-glucose control potato in the experimental design of this study. ^##^ ‘Nadine’ represents the high-glucose control potato in the experimental design of this study.

**Table 2:** The effect of PEF pre-treatment alone (without blanching) on the L*, a* and b* values of fried potato slices (180 °C for 3 min) from ‘Crop77’, ‘Moonlight’, ‘Nadine’, and ‘Russet Burbank’.

Colour Parameters	Non-PEF and No Blanching	PEF Low (1 kV/cm, 50 kJ/kg) without Blanching	PEF High (1 kV/cm, 150 kJ/kg) without Blanching
‘Crop77’			
L*	34.27 ± 2.09 _a_^A^	33.77 ± 2.19 _a_^A^	33.87 ± 2.40 _a_^A^
a*	3.45 ± 1.17 _a_^C^	3.41 ± 1.14 _a_^B^	3.29 ± 0.99 _a_^C^
b*	18.37 ± 2.45 _a_^A^	18.48 ± 2.37 _a_^A^	17.52 ± 2.49 _a_^A^
‘Moonlight’			
L*	29.21 ± 2.48 _ab_^C^	29.75 ± 2.46 _a_^B^	27.96 ± 2.32 _b_^B^
a*	5.37 ± 1.17 _a_^A^	5.05 ± 0.99 _a_^A^	5.52 ± 1.65 _a_^A^
b*	17.47 ± 2.53 _ab_^A^	18.55 ± 2.68 _a_^A^	16.97 ± 2.67 _b_^A^
‘Russet Burbank’			
L*	31.49 ± 1.84 _a_^B^	32.33 ± 2.14 _a_^A^	32.31 ± 1.74 _a_^A^
a*	5.62 ± 1.01 _a_^A^	4.47 ± 1.25 _b_^A^	4.23 ± 0.73 _b_^B^
b*	15.37 ± 1.62 _a_^B^	14.88 ± 1.48 _a_^B^	14.85 ± 1.11 _a_^B^
‘Nadine’			
L*	17.44 ± 3.38 _b_^D^	19.31 ± 2.29 _a_^C^	19.99 ± 3.58 _a_^C^
a*	4.46 ± 1.59 _a_^B^	3.62 ± 0.92 _b_^B^	3.54 ± 1.26 _b_^BC^
b*	9.21 ± 1.99 _a_^C^	8.40 ± 1.31 _a_^C^	8.58 ± 1.38 _a_^C^

The data are presented as mean ± standard deviation (*n* = 15 measurements, 3 measurements from 5 independent potato tubers per treatment). Means in the same row for each cultivar not sharing the same lowercase letter in subscript are significantly different at *p* < 0.05 between untreated and PEF-treated potatoes based on one-way ANOVA and Tukey’s post hoc test. Means in the same column for each colour parameters not sharing the same uppercase letter in superscript are significantly different at *p* < 0.05 between cultivars of the same treatment based on one-way ANOVA and Tukey’s post hoc test.

**Table 3:** The effect of sequential PEF and blanching pre-treatments on the L*, a* and b* values of fried potato slices (180 °C for 3 min) from ‘Crop77’, ‘Moonlight’, ‘Nadine’, and ‘Russet Burbank’.

Colour Parameters	Non-PEF (Blanching Only)	PEF Low (1 kV/cm, 50 kJ/kg) Followed by Blanching	PEF High (1 kV/cm, 150 kJ/kg) Followed by Blanching
‘Crop77’			
L*	35.04 ± 2.26 _b_^A^	37.06 ± 1.80 _a_^A^*	35.78 ± 1.54 _b_^A^*
a*	2.66 ± 1.08 _a_^C^*	1.52 ± 0.46 _b_^D^*	2.23 ± 0.78 _a_^BC^*
b*	17.31 ± 1.87 _a_^A^*	17.59 ± 1.20 _a_^A^	17.39 ± 2.39 _a_^A^
‘Moonlight’			
L*	32.94 ± 2.68 _a_^B^*	33.15 ± 1.77 _a_^B^*	33.72 ± 1.25 _a_^B^*
a*	3.83 ± 1.04 _a_^B^*	3.27 ± 0.85 _b_^B^*	2.78 ± 0.57 _b_^B^*
b*	18.03 ± 1.81 _a_^A^	17.60 ± 2.06 _a_^A^	16.28 ± 1.37 _b_^AB^
‘Russet Burbank’			
L*	33.96 ± 2.07 _b_^AB^*	36.71 ± 0.96 _a_^A^*	36.86 ± 1.12 _a_^A^*
a*	3.85 ± 1.31 _a_^B^*	2.23 ± 0.53 _b_^C^*	2.02 ± 0.60 _b_^C^*
b*	16.11 ± 1.38 _a_^B^*	16.30 ± 0.76 _a_^B^*	15.73 ± 1.00 _a_^B^*
‘Nadine’			
L*	22.34 ± 2.60 _a_^C^*	24.12 ± 2.14 _a_^C^*	23.71 ± 2.96 _a_^C^*
a*	5.37 ± 1.42 _a_^A^*	4.34 ± 1.26 _b_^A^*	5.15 ± 0.61 _ab_^A^*
b*	12.88 ± 2.52 _a_^C^*	11.50 ± 2.32 _a_^C^*	13.03 ± 0.82 _a_^C^*

The data are presented as mean ± standard deviation (*n* = 15 measurements, 3 measurements from 5 independent potato tubers per treatment). Means in the same row for each cultivar not sharing the same lowercase letter in subscript are significantly different at *p* < 0.05 between untreated and PEF-treated potatoes based on one-way ANOVA and Tukey’s post hoc test. Means in the same column for each colour parameters not sharing the same uppercase letter in superscript are significantly different at *p* < 0.05 between cultivars of the same treatment based on one-way ANOVA and Tukey’s post hoc test. * indicates significant difference (*p* < 0.05) in the colour parameters, based on a Student’s *t*-test, due to the blanching treatment when compared to the untreated and PEF-treated potato samples without subjected to a blanching treatment (Table 2).

**Table 4:** Estimated kinetic parameters of time dependence *k* and temperature dependence *E_a_* of changes in lightness (L*) of fried potato slices produced from cultivars ‘Crop77’, ‘Moonlight’, ‘Nadine’ and ‘Russet Burbank’ without and with PEF pre-treatment followed by blanching.

PEF Treatment	Frying Temperature (°C)	*k* (×10^−3^ s^−1^) *	Range of R^2^ for *k* Estimation	*E_a_* (kJ·mol^−1^) **	Range of R^2^ for *E_a_* Estimation
**‘Crop77’**					
Non-PEF	170	0.24 ± 0.04	0.90–0.96	105.53 ± 1.16 _a_^A^	0.90–0.99
(blanching only)	180	0.37 ± 0.03	0.92–0.93		
	190	0.83 ± 0.16	0.93–0.96		
PEF Low	170	0.30 ± 0.07	0.84–0.91	86.78 ± 1.19 _c_^A^	0.99–1.00
(1 kV/cm,	180	0.49 ± 0.12	0.93–0.98		
50 kJ/kg) + blanching	190	0.82 ± 0.20	0.82–0.98		
PEF High	170	0.22 ± 0.03	0.86–0.87	102.41 ± 0.32 _b_^A^	1.00–1.00
(1 kV/cm,	180	0.40 ± 0.07	0.89–0.98		
150 kJ/kg) + blanching	190	0.72 ± 0.11	0.95–0.98		
**‘Moonlight’**					
Non-PEF	150	0.86 ± 0.05	0.89–0.93	31.73 ± 1.03 ^D^	0.95–1.00
(blanching only)	170	1.27 ± 0.12	0.94–0.96		
	190	1.87 ± 0.17	0.93–0.95		
PEF Low	150	0.77 ± 0.14	0.87–0.94	32.82 ± 0.23 ^D^	0.99–0.99
(1 kV/cm,	170	1.22 ± 0.22	0.88–0.97		
50 kJ/kg) + blanching	190	1.72 ± 0.30	0.88–0.95		
PEF High	150	0.84 ± 0.03	0.81–0.97	31.09 ± 0.81 ^C^	0.91–1.00
(1 kV/cm,	170	1.23 ± 0.17	0.50–0.86		
150 kJ/kg) + blanching	190	1.81 ± 0.03	0.87–0.95		
**‘Russet Burbank’**					
Non-PEF	150	0.94 ± 0.13	0.85–0.92	45.37 ± 0.85 _a_^B^	0.96–1.00
(blanching only)	170	1.63 ± 0.21	0.84–0.95		
	190	2.87 ± 0.37 _a_	0.87–0.90		
PEF Low	150	0.76 ± 0.07	0.86–0.86	35.37 ± 0.76 _b_^C^	0.97–0.98
(1 kV/cm,	170	1.16 ± 0.26	0.83–0.83		
50 kJ/kg) + blanching	190	1.82 ± 0.15 _b_	0.80–0.93		
PEF High	150	0.66 ± 0.23	0.85–0.98	31.80 ± 1.08 _c_^C^	0.91–1.00
(1 kV/cm,	170	1.11 ± 0.43	0.85–0.90		
150 kJ/kg) + blanching	190	1.45 ± 0.53 _b_	0.81–0.91		
**‘Nadine’**					
Non-PEF	150	1.09 ± 0.41	0.82–0.89	41.15 ± 1.87 ^C^	0.99–1.00
(blanching only)	170	1.93 ± 0.66	0.84–0.93		
	190	2.96 ± 0.99	0.91–0.96		
PEF Low	150	0.95 ± 0.45	0.84–0.97	39.92 ± 1.12 ^B^	0.88–0.96
(1 kV/cm,	170	1.69 ± 0.99	0.75–0.81		
50 kJ/kg) + blanching	190	2.50 ± 1.12	0.92–0.94		
PEF High	150	1.15 ± 0.31	0.84–0.88	38.85 ± 1.46 ^B^	0.87–1.00
(1 kV/cm,	170	2.06 ± 0.63	0.88–0.89		
150 kJ/kg) + blanching	190	2.95 ± 0.72	0.82–0.93		

* The rate of changes in L* (*k*) is presented as average of estimated kinetic parameter ± standard deviation of the estimates from three independent frying kinetic experiments (where each experiment involved a batch of 8–10 potatoes). Estimated *k* values within the same cultivar and frying temperature not sharing the same lowercase letter in subscript are significantly different at *p* < 0.05 between non-PEF and PEF-treated potatoes based on one-way ANOVA and Tukey’s post hoc test. ** Activation energy (*E_a_*) is presented as average of estimated kinetic parameter ± standard deviation of the estimates from three independent frying kinetic experiments (where each experiment involved a batch of 8–10 potatoes). Estimated *E_a_* values within the same cultivar not sharing the same lowercase letter in subscript are significantly different at *p* < 0.05 between non-PEF and PEF-treated potatoes based on one-way ANOVA and Tukey’s post hoc test. Estimated *E_a_* values within the same treatment group not sharing the same uppercase case letter in superscript are significantly different at *p* < 0.05 between cultivars based on one-way ANOVA and Tukey’s post hoc test.

## Data Availability

The datasets generated for this study are available on request to the corresponding author.

## Supplementary Materials

The following are available online at https://www.mdpi.com/article/10.3390/foods10102307/s1, File S1: Determination of the content of total starch, amylose, glucose, total amino acids, and dry matter of the tubers.
