# Supplementary material for: Kinetics of Colour Development during Frying of Potato Pre-Treated with Pulsed Electric Fields and Blanching: Effect of Cultivar
Source: Foods. 2021 Sep 28;10(10):2307. doi: 10.3390/foods10102307 (PMC8535209; doi:10.3390/foods10102307)
Supplement: Supplementary file 1 [file foods-10-02307-s001.zip › foods-1379749-supplementary.pdf]

foods

Supplementary Material

Kinetics of Colour Development during Frying of Potato Pre-Treated with Pulsed Electric Fields and Blanching: Effect of Cultivar

Setya Budi Muhammad Abduh, Sze Ying Leong, Chun Zhao, Samantha Baldwin, David J. Burritt, Dominic Agyei, Indrawati Oey*

Correspondence: Department of Food Science, University of Otago, Dunedin 9054, New Zealand

indrawati.oey@otago.ac.nz; Tel.: +64-3-479-8735

File S1:

Determination of the content of total starch, amylose, glucose, total amino acids, and dry matter of the tubers

(A) Determination of total starch content of potato tubers

The analysis of total starch of all four potato cultivars was carried out using a colorimetric assay (based upon enzymatic reaction of α-amylase/amyloglucosidase) from Megazyme (Bray, County Wicklow, Ireland). Five hundred milligrams of ground-frozen potato sample were added to 500 µL of 80% (v/v) ethanol. The dispersion was then vortexed and placed on a magnetic stirrer followed by the addition of 1 mL of 2 M potassium hydroxide (Thermo Fischer Scientific Australia Pty Ltd, Melbourne, Australia) to dissolve any resistant starch in the sample. The mixture was stirred for 20 min in an ice water bath using a magnetic stirrer. Afterwards, 4 mL of 1.2 M sodium acetate buffer pH 3.8 prepared using glacial acetic acid purchased from Ajax Finechem (Melbourne, Australia) and sodium hydroxide from Merck (Darmstadt, Germany) was added to neutralise the pH of the mixture. Then, 50 µL of 3000 U/mL heat-stable α-amylase from *Bacillus licheniformis* (Megazyme, Bray, County Wicklow, Ireland) and 50 µL of 3300 U/mL amyloglucosidase from *Aspergillus niger* (Megazyme, Bray, County Wicklow, Ireland) were added, followed by incubation at 50°C in a water bath for 1 hour with an intermittent vortex mixing every 10 min to hydrolyse insoluble starch into soluble branched dextrin and to hydrolyse the dextrin into D-glucose. The mixture was then brought up to 40 mL volume with deionised water (Sartorius Stedim Biotech Arium 611UV, Goettingen, Germany), vortexed and centrifuged (Beckman GPR, Indianapolis, USA) at 1613*g* for 10 min. Fifty microlitres of the supernatant were then added to 1.5 mL of glucose oxidase and peroxidase (GOPOD) reagent (Megazyme, Bray, County Wicklow, Ireland) and heated at 50°C for 20 min. Absorbance was measured at 510 nm and 20°C using a UV–VIS spectrophotometer (Ultraspec 3300 Pro Amersham Biosciences, Sweden) with a D-glucose solution (1 mg/mL) (Megazyme, Bray, County Wicklow, Ireland) as the external standard. Results were expressed as milligram starch per gram potato dry weight, using a conversion factor of 0.9, which is generally calculated from the molecular weight of starch monomer divided by the molecular weight of glucose (162/180 = 0.9). The total starch measurement for each potato cultivar was conducted in triplicate.

(B) Determination of amylose content

The amylose content of potato was determined using an amylose-iodine binding complex assay (Kaufman, Wilson, Bean, Herald, & Shi, 2015). Initially, a 50 mg ground frozen potato sample was transferred into a 2 mL tube, and 1 mL of 90% (v/v) dimethyl sulfoxide (DMSO) (Fischer Scientific, Loughborough, UK) was added. Subsequently, the sample was heated in a water bath at 95°C for 60 min with regular vortex mixing at a 10 min interval. After cooling down to room temperature, the sample was centrifuged at 2300*g* (Thermo IEC Micromax, Massachusetts, USA) for 10 min. One hundred microlitres of supernatant was added with 100 µL DMSO containing 3.4 g/L iodine (I_2) and then mixed for 2 min. Twenty microlitres of the mixture were transferred into a 96-well plate, in duplicate, followed by addition of 180 µL of deionised water, and mixed for 2 min. The absorbance was read at 620 and 510 nm against a control blank (mixture of 100 µL DMSO with 100 µL I_2-containing DMSO) using a microplate reader (Synergy 2, BioTek Instruments, Inc., Winooski, VT, USA). A standard calibration curve was prepared from mixtures of 5 mg/mL amylose (Sigma, St Louis, USA) and 5 mg/mL amylopectin (Sigma, St Louis, USA) in DMSO with a varying ratio. The amylose content was calculated as the difference of absorbance at 620 and 510 nm and the result were expressed as mg/g of dry sample. The measurement for each potato cultivar was conducted in triplicate.

(C) Determination of glucose content

One gram of ground-frozen sample was transferred into a 15 mL tube, followed by the addition of deionised water (2–12 mL, depending on the cultivar). The mixture was vortexed for 10 s at high speed and centrifuged at 1613 *g* and room temperature (20–22°C) (Beckman GPR, Indianapolis, USA) for 10 min. One hundred microlitres of supernatant (potato extract) were removed and diluted in 100 µL sodium acetate buffer (10 mM, pH 4.5), followed by addition of 1.5 mL glucose oxidase and peroxidase (GOPOD) reagent (Megazyme, Bray, County Wicklow, Ireland). The mixture was incubated in a water bath (Grant Instruments, Cambridge, UK) at 50°C for 20 min and the final absorbance was read at 510 nm UV/visible spectrophotometer (Ultraspec 3300 Pro, Amersham Biosciences, Sweden) against the reagent blank. The reagent blank consisted of deionised water, which was reacted with GOPOD reagent under similar conditions as the potato extract. In order to quantify the amount of glucose in the potato extract, a D-glucose standard (1 mg/mL) (Megazyme, Bray, County Wicklow, Ireland) was used and reacted with GOPOD reagent under similar conditions as the potato extract. The resulting glucose content was expressed as milligram per gram potato dry weight. The measurement for each potato cultivar was conducted in triplicate.

(D) Determination of total amino acids content

For the measurement of amino acids, the potato samples were extracted as follows: 1 g of frozen potato powder was mixed with 20 mL 0.01 M hydrochloric acid (Merck, Darmstadt, Germany). The mixture was then homogenised on ice water bath using an Ultra Turrax homogenizer (IKA T25, Staufen, Germany) at 25000 rpm for 5 min, followed by centrifugation at 1613*g* and room temperature (Beckman GPR, Indianapolis, USA) for 20 min. The supernatant (or potato extract) was aliquoted and kept frozen at –20°C until analysis within 1 week.

The total amino acids in each potato extract were analysed using EZ:faast amino acid kit (Phenomenex, Aschaffenburg, Germany), which consists of a solid-phase extraction step, followed by derivatisation and a liquid/liquid extraction. The frozen aliquot was thawed at room temperature and centrifuged at 4600*g* (Thermo IEC Micromax, Massachusetts, USA). One hundred microlitres of potato extract were derivatised according to the instructions from the EZ:faast amino acid kit and 2 µL of derivatised sample were injected into a GC-FID system (Agilent 7683, Agilent Technologies, California, USA) at 250°C using an autosampler. By using helium gas as a carrier, the samples were passed through a Zebron ZB-AAA column (Phenomenex, Aschaffenburg, Germany) at a constant flow at 1.5 mL/min in a 15:1 split ratio. With an oven programme of 35°C/min heating rate from 80 to 320°C, FID detection was carried out at 320°C. For each potato cultivar, six samples were injected in duplicate. Amino acids were quantified using a calibration curve, with standards at concentrations ranging from 20 to 400 nmol/mL and are reported in µmol/g on a dry weight basis. The total amino acid content was estimated as the sum of concentration for each amino acid found in the potato sample.

(E) Determination of moisture content and dry matter

The moisture content of potato samples was determined gravimetrically. One gram of potato was dried in an oven (Qualtex, Andrew Thom Limited, Sydney, Australia) at 95 ± 5°C for at least 48 h or until a constant weight was achieved. The dry matter was used for the basis of calculation for total starch, amylose, glucose, and amino acids content for each sample.

Reference:

Kaufman, R.C.; Wilson, J.D.; Bean, S.R.; Herald, T.J.; Shi, Y.C. Development of a 96-well plate iodine binding assay for amylose content determination. *Carbohydrate Polymers* **2015**, *115*, 444-447.
